# Motion-induced distortion of shape

**DOI:** 10.1167/jov.23.12.10

**Published:** 2023-10-30

**Authors:** Nika Adamian, Stuart Anstis, Patrick Cavanagh

**Affiliations:** 1 School of Psychology, University of Aberdeen, Old Aberdeen, SC, UK; 2 Department of Psychology, University of California, San Diego, La Jolla, CA, USA; 3 Department of Psychology, Glendon College, CVR York University, Toronto, ON, Canada

**Keywords:** vision, motion, shape

## Abstract

Motion, position, and form are intricately intertwined in perception. Motion distorts visual space, resulting in illusory position shifts such as flash-drag and flash-grab effects. The flash-grab displaces a test by up to several times its size. This lets us use it to investigate where the motion-induced shift operates in the processing stream from photoreceptor activation to feature activation to object recognition. We present several canonical, highly familiar forms and ask whether the motion-induced shift operates uniformly across the form. If it did, we could conclude that the effect occurred after the elements of the form are bound. However, we find that motion-induced distortion affects not only the position, but also the appearance of briefly presented, canonical shapes (square, circle, and letter T). Features of the flashed target that were closest to its center were shifted in the direction of motion more than those further from its center. Outline shapes were affected more than filled shapes, and the strength of the distortion increased with the contrast of the moving background. This not only supports a nonuniform spatial profile for the motion-induced shift but also indicates that the shift operates before the shape is established, even for highly familiar shapes like squares, circles, and letters.

## Introduction

Successful interaction with a dynamic environment requires rapid integration of the form, motion and position of objects and this integration often leads to dramatic interactions. For instance, motion biases the perceived location of stationary (De Valois & De Valois, 1991; Ramachandran & Anstis, 1990) and moving (Freyd & Finke, 1984; Frohlich, 1923) stimuli, as well as stimuli briefly presented some distance away from the motion (Whitney, 2002; Whitney & Cavanagh, 2002). One of the largest known motion-induced position shifts occurs when an object is flashed on top of the moving background as it reverses direction (flash-grab, Cavanagh & Anstis, 2013). The flash-grab effect shifts the perceived the position of the flashed object in the direction of motion after the reversal by up to several degrees of visual angle.

A number of studies indicate that motion not only shifts the perceived position of objects, but also alters their appearance, such as their size and shape (Ansbacher, 1944; Anstis, Stürzel, & Spillmann, 1999; Zanker, Quenzer, & Fahle, 2001). Often these perceptual deformations accompany motion-induced position shifts. For instance, Shim and Cavanagh (2004) and Shim and Cavanagh (2006) reported that apparent motion can shift the perceived position of a stationary flash presented near the motion path. Khuu, Phu and Khambiye (2010) using similar conditions revealed that concurrently with the position shift, apparent motion distorts the shape of an object presented along its path, so that the shape appears elongated along the axis of motion. Another example of simultaneous position shift and deformation is found in the stationary Gabor pattern that contains sinusoidal grating in motion. The perceived position of such a stimulus is biased towards the direction of motion (De Valois & De Valois, 1991) and its perceived shape is elongated, with the leading edge extended in the direction of motion (Tsui et al., 2007). Zanker et al. (2001) reported that a straight line in motion appeared bent in the center toward the direction of motion, with its endings trailing the central segment.

The purpose of the current study is to determine whether the parts of the briefly presented target are shifted equally by the motion or whether certain parts of the stimulus will be shifted more strongly than others. If the motion-induced shift is uniformly applied to the different parts of an object, the spatial relationship between the parts will be preserved and the target will appear shifted, but not distorted. This is the case for tests flashed around the time of a saccade (Matsumiya & Uchikawa, 2001) where the tests are shifted toward the saccade target (saccadic compression) but the shapes of the tests themselves are undistorted, suggesting that the location shift is applied after the object shape has been established. Alternatively, the amount of illusory shift could depend on the location of features in the test (as in case of Zanker et al., 2001) so that the target will appear both shifted and distorted.

**Figure 1.**
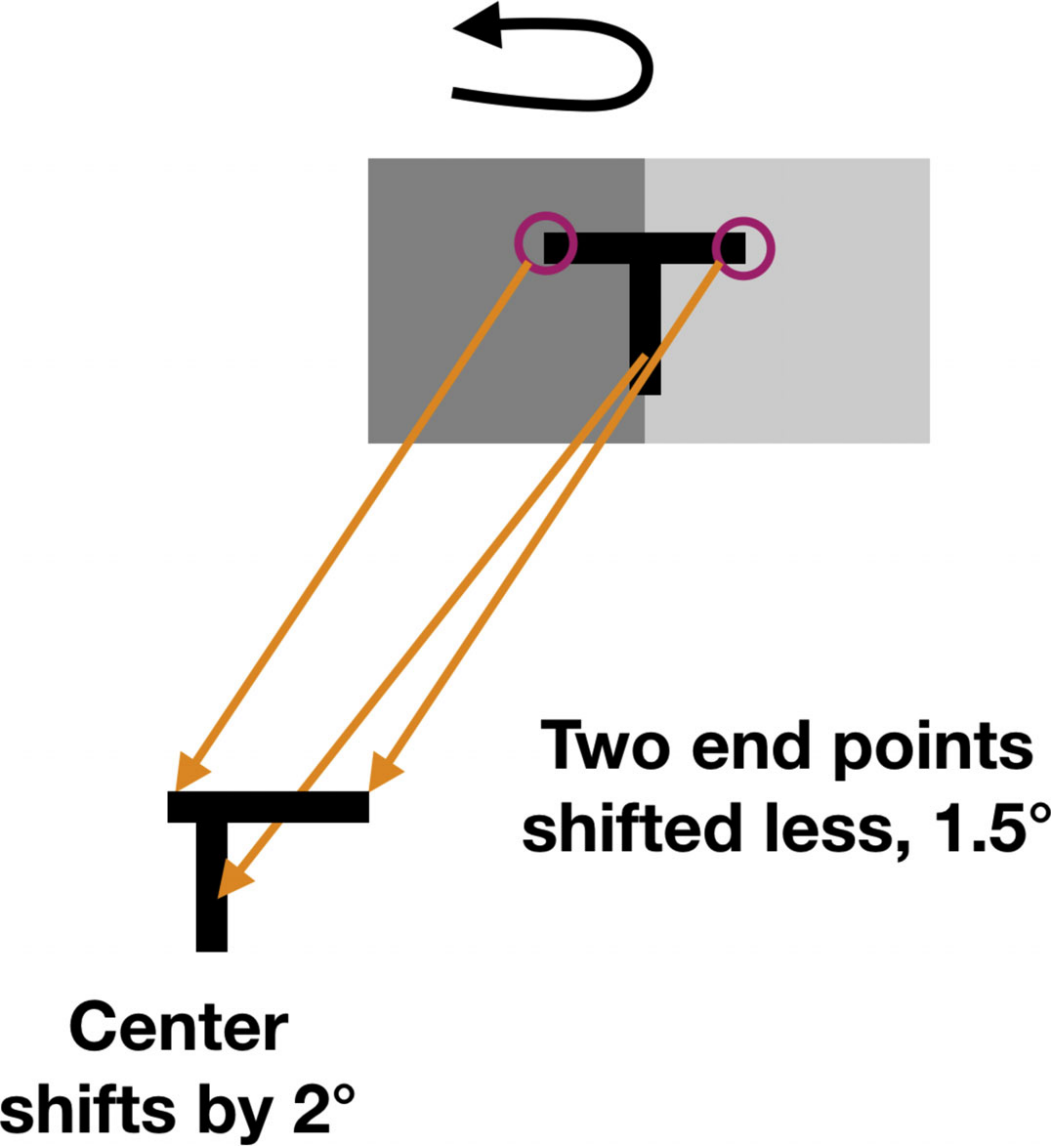
Spatially nonuniform shift. If the flash grab effect is larger at the contrast border than immediately to its left and right, the central stem of the T will be shifted leftward relative to the endpoints of the top bar, producing an asymmetrical shape.

There is evidence that the magnitude of the flash grab effect is spatially local, being maximum at the edge of the moving sector and dropping off rapidly away from that location (Cavanagh & Anstis, 2013, their Figure 8). To demonstrate this difference perceptually, they flashed a large plus sign “+” on a rotating sectored pattern where the vertical arm was aligned with a contrast edge while the horizontal arm fell in the center of a sector (https://cavlab.net/Demos/ShapeDistortion/Wonky), far from the edges. This created a large shift for the arm aligned to the sector's edge and little or no shift for the other arm because of its distance from the edge. As a result, the right angle was significantly distorted. In the case of the shapes that we present here, if this spatial nonuniformity of the shift operates within an object, the middle of the flashed stimulus should be shifted more than its left or right edges, creating an asymmetry in its perceived shape (Figure 1) that is similar to the distortion that may be seen in Supplementary Movie S1.

## Method

### Participants

Ten observers (6 female, 1 author, mean age 26 ± 3) participated in the experiment. All observers had normal or corrected-to-normal vision. Study protocols were approved by the Université Paris Descartes Review Board in accordance with French regulations and the Declaration of Helsinki. Informed consent was obtained in writing prior to participation. Participants were reimbursed for their time. All observers (except for one author) were naïve to the purposes of the experiment and were not familiar with the flash-grab illusion.

### Apparatus and stimuli

Participants were seated in a quiet, dimly lit room. The participant's head was positioned on a chin rest with a forehead stabilizer at 130cm from the projection screen that subtended 60° by 34° of visual angle (dva). Stimuli were displayed with a PROPixx projector (VPixx Technologies, Saint-Bruno-de-Montarville, QC, Canada) at 120 Hz. The experiment was programmed and presented with MATLAB using Psychtoolbox (Brainard, 1997) and Eyelink toolbox (Cornelissen, Peters, & Palmer, 2002) and was run on an Apple computer. The right eye was monitored using an Eyelink 1000 Plus desktop mount (SR Research, Kanata, ON, Canada ) at 1000 Hz.

All stimuli were presented against a mid-gray background. A black fixation point (0.25 dva in diameter) was always presented in the middle of the screen. The stimulus consisted of an annulus (7.5 dva inner radius, 10 dva outer radius) divided into four alternating light and dark sectors. The contrast of the annulus varied from trial to trial and could take one of the five values: 5%, 10%, 20%, 40%, 80%. The annulus rotated at a speed of 135°/s and reversed direction every 90° (80 frames, 666 ms). The polarity of the annulus and the starting direction of motion were counterbalanced within each condition. On every second reversal (two reversals per cycle), motion stopped for 50 ms (six frames) and a green ellipse (2.5° wide and 4° long) with a black target on it was presented on the top sector edge within the annulus. The timing of the reversal was jittered from trial to trial so that the position of the sector edge and its flashed target at the reversal point was varied over ±23.5° of rotation. There were five conditions depending on the shape of the target: filled rectangle, outline rectangle, filled ellipse, outline ellipse, and T-shape.

**Figure 2.**
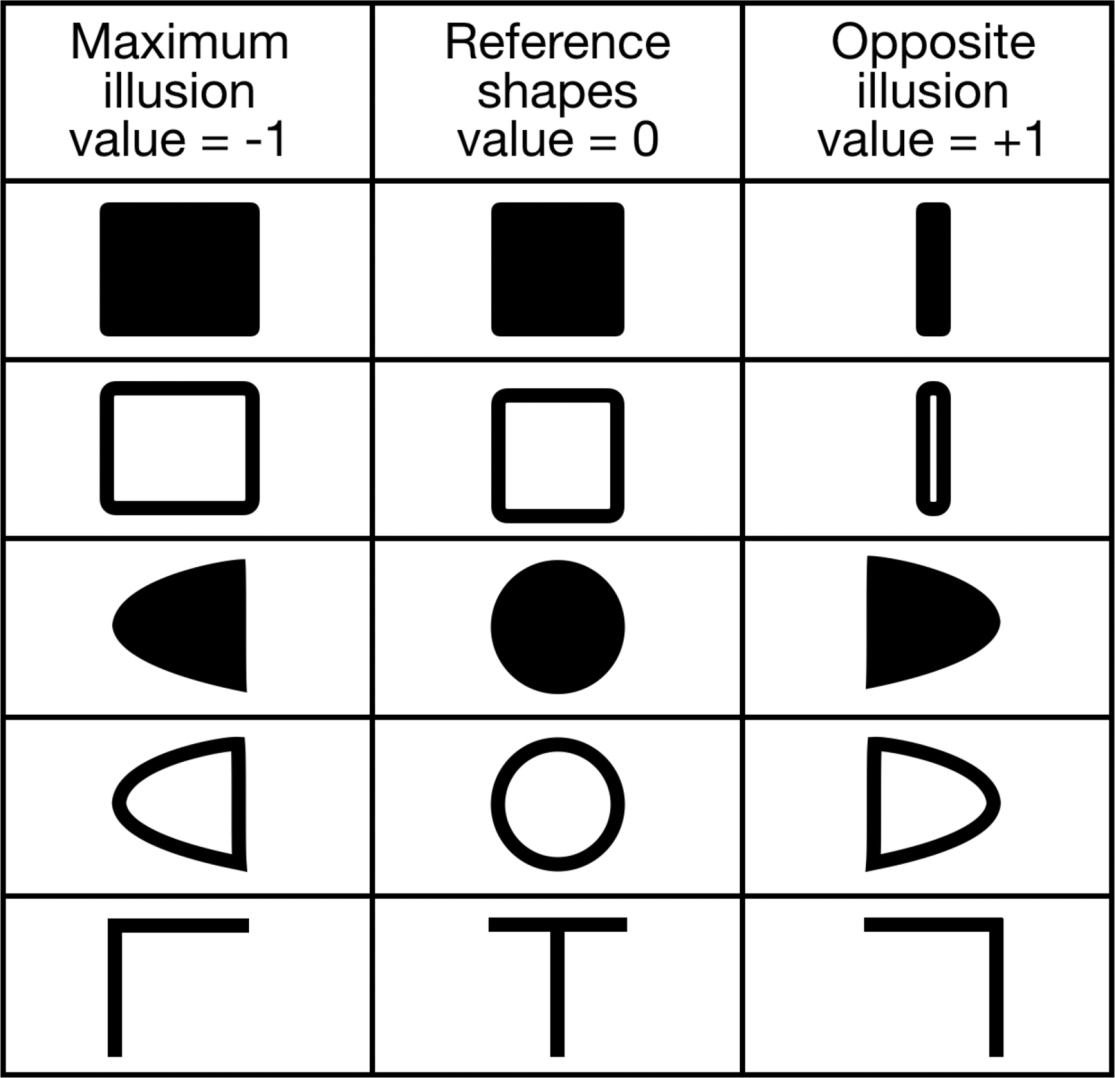
Target shapes and their adjustable versions. Participants adjusted the test form between the two extremes until the flashed test appeared to match the reference shape. The middle column shows the reference shapes, and these were scored 0 (no illusion) if participants reported that this shape matched the reference shape. The left column shows the exaggerations of the illusory distortions seen in the pilot study and were scored −1 if chosen. The right column shows the reverse of the left column, and these were scored +1, representing a very strong illusion that required an extreme opposite distortion to cancel.

Based on pilot observations of the various distortions for these stimuli, we generated parametric shape changes that might cancel these distortions (Figure 2, see demonstration movies at https://cavlab.net/Demos/ShapeDistortion/DemoAll5). The following distortions were noted: the square appeared to expand in width, the circle expanded to become egg-shaped blunted at leading edge (in the direction of the motion after the reversal), and the vertical stem of the T shifted relative to the top in the direction of the motion after the reversal. The flashed shape has no motion itself but is displaced as if it were in motion with the background after the reversal (Cavanagh & Anstis, 2013). The “leading edge” is then the edge of the test shape that is farthest from the background contour in the direction of the motion after the reversal. We exaggerated these distortions to generate one end of the adjustment scale and then mirrored these to create a full range of adjustments that should at some point restore the stimuli to the canonical shapes: square, circle, and T. The reference shape was in the middle of the adjustment range and was separated from each end of the range by 40 steps. The illusion end of the shapes was given a score of −1 (left column in Figure 2), the reference shape in the middle, 0 (middle column Figure 2), and the shapes with the maximum reversed distortions, a value of +1 (right column, Figure 2).

Contours were always 0.2 dva thick. Rectangles were 1.5 dva high and had adjustable width (from 0.5 dva to 2.5 dva). Elliptical shapes were created by merging two complementary halves of ellipses with the same vertical axis (1.5 dva) and variable horizontal axes. The width of the resulting asymmetric “egg”-shape was always 1.5 dva, and the degree of asymmetry was adjustable. Finally, a T shape was constructed from a 1.5 dva wide horizontal segment and a 1.5 dva long vertical segment. The horizontal segment was fixed in the middle of the green background ellipse, while the vertical segment could be moved left and right.

### Procedure

Trials were self-paced. Before each trial, the reference shape (symmetrical T, circle, or square, see the middle row of Figure 1) was shown in the middle of the screen. During the trial observers had to fixate in the middle of the screen and adjust the shape presented during motion reversals to match the reference shape shown before by pressing the arrow keys on a keyboard.

**Figure 3.**
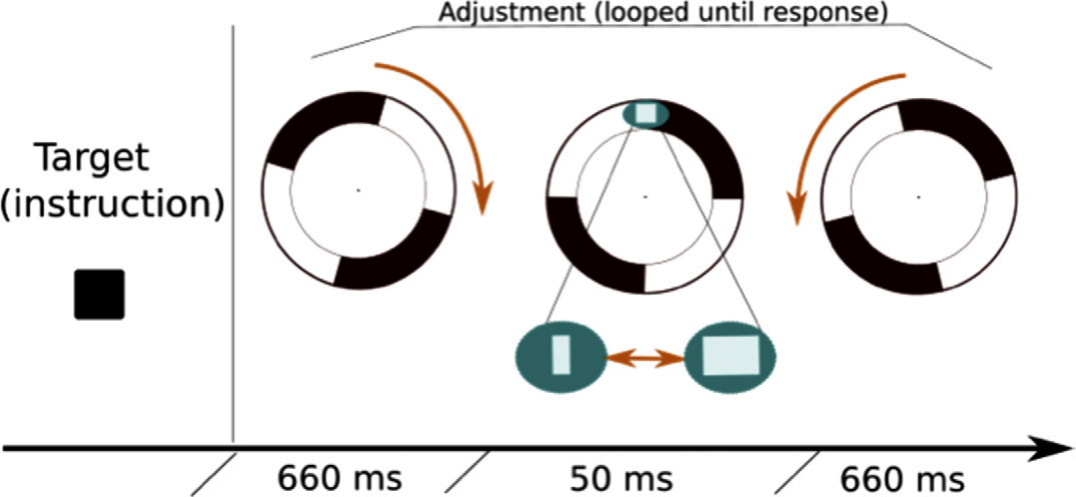
Trial sequence. Each trial started with the presentation of the target shape. The stimulus was then presented in motion, with the adjustable target briefly flashed on every second reversal.

The sectored annulus rotated back and forth continuously with the target present once each cycle until the response was made (Figure 3). The initial appearance of the shape was randomly drawn from all its possible states. Eye position was controlled 300 ms before and after the presentation of the target. If the gaze was detected outside the fixation window during this period (1 dva around fixation), the trial was aborted and replaced later. Although targets were on screen only briefly during reversals, participants could keep changing the shape in between presentations, evaluating the result of the adjustment when the target next appeared. Responses were centered and recoded so that 0 corresponded to the reference shape, negative responses corresponded to the distortion in the direction of motion before the flash, and positive responses—to distortion in the direction of motion after the flash (i.e. in the flash-grab direction).

Two factors were manipulated in the experiment: target shape and background contrast with five levels of each and eight repetitions for a total of 200 trials. The experiment was carried out in two sessions of approximately one hour each. Trials from different contrast and shape conditions were presented in a random order.

## Results

**Figure 4.**
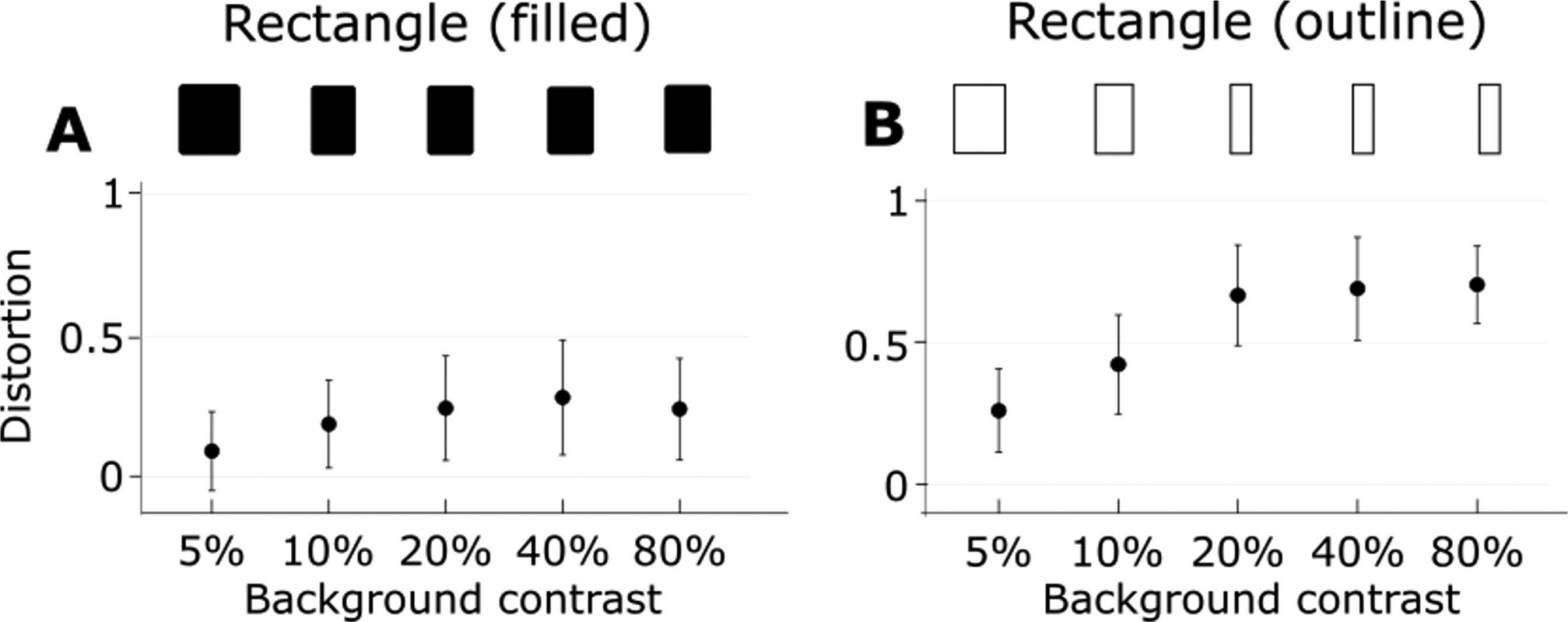
Distortion of rectangular shapes as a function of contrast. The top row represents the shape that was perceived as a square in each condition. Error bars represent ±1 SEM.

**Figure 5.**
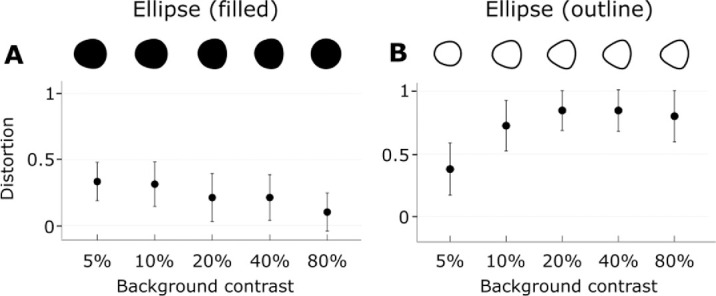
Distortion of elliptical shapes as a function of contrast. The top row represents the shape that was perceived as a circle in each condition. Error bars represent ±1 SEM.

**Figure 6.**
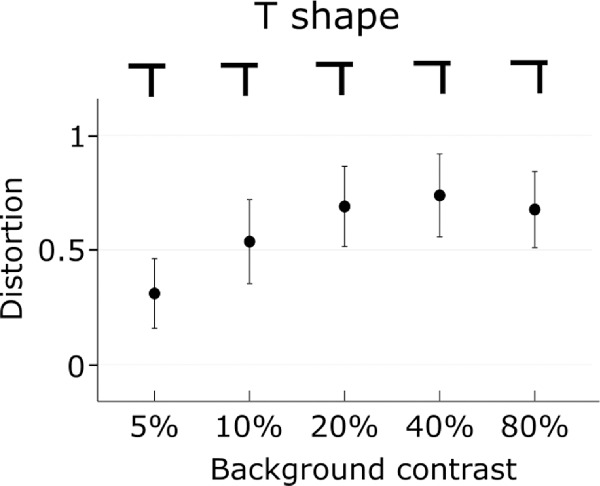
Distortion of the T as a function of contrast. The top row represents the shape that was perceived as a symmetrical T in each condition. Error bars represent ±1 SEM.

The analysis was aimed at two questions: (a) the extent to which each of the shapes was distorted by motion, and (b) whether this distortion was mediated by the background contrast. Trials from different shape conditions were analyzed separately. Using single-sample *t*-tests, the average responses across all contrasts were tested against the zero point to determine whether the shape was subject to distortion. Means of the responses were then subjected to a one-way repeated measures analysis of variance with background contrast as a factor. Data for each shape and contrast condition are presented on Figures 4 to 6.

### Rectangles

Both filled and outline rectangles were significantly expanded by motion—they required test rectangles narrower than a square to appear square (Figure 4; Filled: *t_(9)_* = 4.65, *p* = 0.001; Outlined: *t_(9)_* = 16.02, *p* < 0.001). The distortion increased as a function of background contrast for outline (*F_(4,36)_* = 3.49, *p* = 0.02, *η^2^* = 0.28) but remained at a moderately low level for filled rectangles independently of contrast (*F_(4,36)_* = 0.25, *p* = 0.91, *η^2^* = 0.03).

### Ellipses

Both filled and outline ellipses were distorted by motion such that the side of the ellipse leading after the reversal was blunter, and the opposite side was pointier than the physically presented shape, requiring that the target be pointier on the leading side to appear as a circle (Figure 5; Outlined: *t_(9)_* = 14.32, *p* < 0.001; Filled: *t_(9)_* = 7.84, *p* < 0.001). Note that the test shapes have no physical motion but are assumed to take on the motion of the background after the flash (Cavanagh & Anstis, 2013) and so have a leading and trailing edge. Outline ellipses were distorted more than filled ellipses (*t_(9)_* = 8.88, *p* < 0.001) and the analysis of variance showed a linear increase of distortion with contrast for outline ellipses only (Outlined: *F_(4,36)_* = 3.39, *p* = 0.02, *η^2^* = 0.27; Filled: *F_(4,36)_* = 1.48, *p* = 0.23, *η^2^* = 0.14).

### T shape

The vertical segment of the T shape was shifted toward the end of the horizontal segment, which was leading after the reversal, requiring the reverse of this shift to appear as a symmetrical T-shape (Figure 6; *t_(9)_* = 12.17, *p* < 0.001). There was a significant linear increase of distortion with contrast (*F_(4,36)_* = 3.28, *p* = 0.02, *η^2^* = 0.26).

Overall, we observed distortions of all briefly presented shapes. The distortion appeared to be driven principally by a greater shift of the central features of the shapes relative to the outer features, generating an expansion on the trailing edge and a compression on the leading edge. These distortions were stronger for contours than for filled shapes and were generally stronger at higher contrasts of the moving edge.

## Discussion

**Figure 7.**
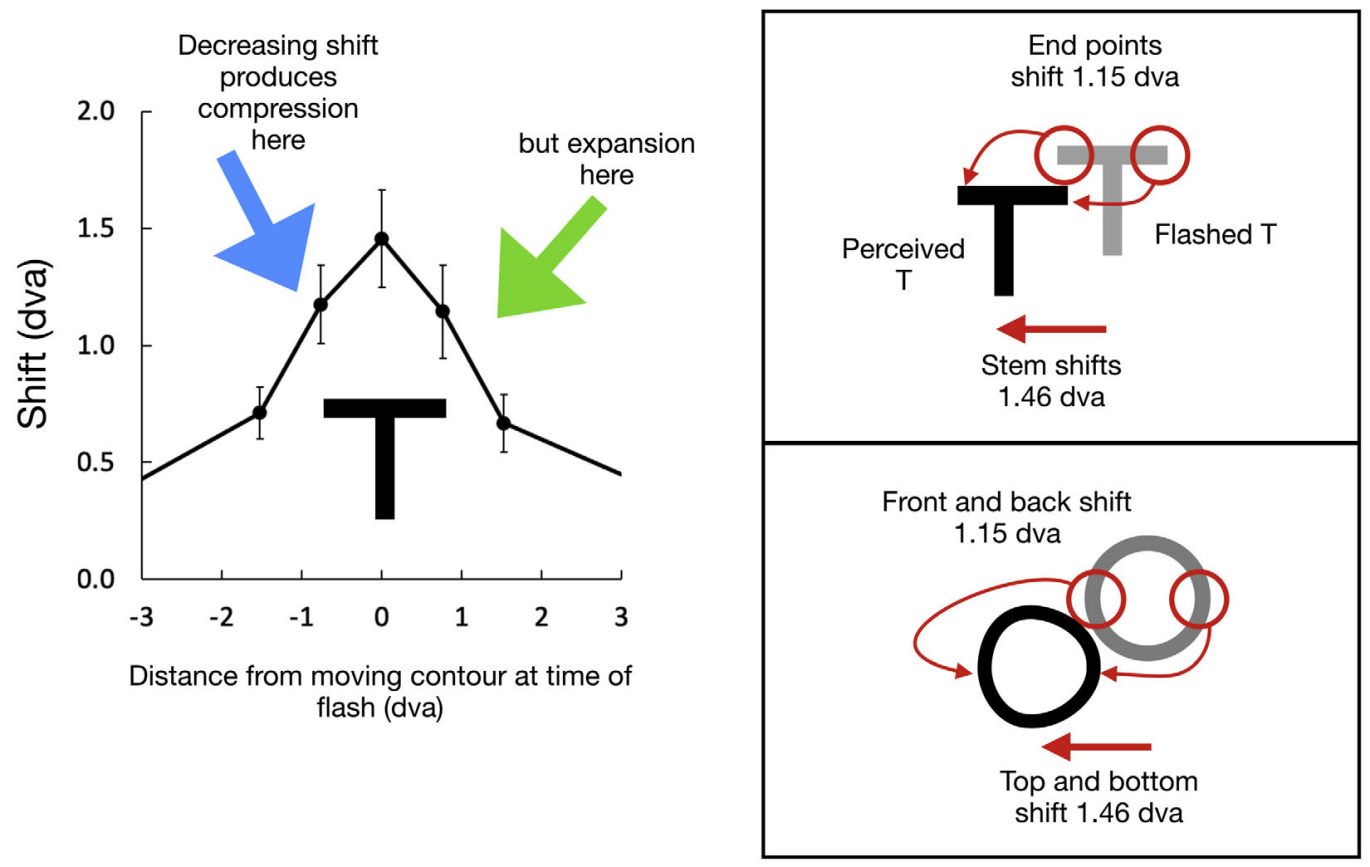
The central part of the shift data from Cavanagh and Anstis (2013) is shown on the left, converted to dva. Their test probe was a compact green flash, but our T-shape is shown here at the appropriate size. Notice that the shift values would be larger at the end points of the T than at its stem. On the right, the effect of these different shifts is shown for the T-shape on top and the circle on the bottom. The distortions in the experiment are in the directions predicted here but larger.

The experiment showed that when a briefly presented target is perceptually shifted by the background motion, it is also distorted. The observed distortions are consistent with a spatial variation in the magnitude of the shift which leads to an asymmetric elongation (Figure 7). The distortions predicted from the earlier shift data (Cavanagh & Anstis, 2013) are in the right direction for the T and circle although smaller than we observed here. This suggests that if this nonuniform shift profile is the source of the distortions, it must be more accentuated for our conditions, which differed in rotation speed and the contrast of moving contour from the earlier study. This accentuated profile would also expand a rectangle into a square once the compression on the leading edge was extreme enough to flatten the leading half while the trailing half was expanded. These explanations for the distortions are plausible but tentative until further tests can examine the effects more directly.

Interestingly, in most cases, the strength of the perceived distortion depended on the background contrast, showing a profile different from that reported for the flash-grab itself (Cavanagh & Anstis, 2013). In this earlier study, the size of the flash- grab illusion increased with the contrast of the moving texture up to around 5% contrast, where it saturated. The saturation was linked to the function of magnocellular pathway, which exhibits high contrast gain but saturates at fairly low contrast (Derrington & Lennie, 1984; Snippe, 1998). In comparison, distortions of shape here showed a linear increase up to 20% to 40% contrast, suggesting that the coding of local spatial configurations that is affected by motion relies at least partly on a different processing route.

The spatial gradient of shift was most evident for the outline shapes and less so for filled shapes (Figures 4 and 5). This suggests that the shift may operate on any available low spatial frequencies and carry associated high spatial frequency details along with it. With the relative absence of low spatial frequencies for the outline figures, the local variations in the degree of shift were more noticeable.

In accounting for the motion-induced position shifts such as the flash-grab, a major issue is to determine the stage of visual processing where the interaction between the motion and position occurs. A number of studies demonstrate that motion can influence coding of position early in the processing stream (Fu, Shen, Gao, & Dan, 2004; Hogendoorn, Verstraten, & Cavanagh, 2015; Kosovicheva et al., 2012). However, other studies suggest a much higher-level locus of interaction (Cavanagh & Anstis, 2013; de Vito et al., 2015; Linares, López-Moliner, & Johnston, 2007; Tse, Whitney, Anstis, & Cavanagh, 2011). In this study, we demonstrated that motion-induced shifts operate to a different degree across the extent of an object (Figure 7), which points to an early interaction between motion and position at a site before the establishment of the object's shape. This earlier site differs from the results seen for motion itself when it affects position. Specifically, an array of Gabors produces position shifts that depend on the global motion of the array after the local motions are bound together (e.g., Rider, McOwan, & Johnston, 2009) rather than showing independent shifts across locations as found for the shape distortions here.

Eye movements are a well-known source of perceived distortions and displacements. When a bar is briefly displayed immediately before the saccade, its position is shifted toward the saccade goal if it is between the initial fixation and the saccade target or in the opposite direction if the bar is presented beyond the saccade target. This is known as saccadic compression (Morrone, Ross, & Burr, 1997; Ross, Morrone, & Burr, 1997). When multiple squares are presented, their spacing appears to be dramatically compressed but, importantly, the width of each square itself is unchanged (Matsumiya & Uchikawa, 2001). This absence of shape distortion in the presence of large position shifts suggests that, unlike the motion-induced distortions seen here, the perisaccadic position shifts act on individual objects at a level beyond the establishment of shape.

According to our findings, even the elements of a highly familiar shapes like circles and squares are distorted by motion, demonstrating that canonical shape does not protect against this early, motion-induced mislocalization. The results show that motion-induced position shifts must act before shapes are established. Our proposal for a nonuniform profile of spatial shifts is promising as an explanation but would need more direct tests to be confirmed.

## Supplementary Material

Supplement 1

## Supplementary material

Supplementary Movie S1. Shape distortion. While fixating the central dot, pay attention to the shape that appears at the top at the moment the motion reverses. It is a T with its top horizontal line segment symmetrically placed on top of the vertical stem. However, it may appear to be offset with the stem nearer the left end of the top line making more of a г shape. Click here to see the movie, then click on the movie to start it, https://cavlab.net/Demos/ShapeDistortion/Movie1.
